# Trends in the Academic Credentials of Matched Dermatology Residency Applicants

**DOI:** 10.7759/cureus.12411

**Published:** 2020-12-31

**Authors:** Ledibabari M Ngaage, Shealinna Ge, Cynthia Gao, Michael Ha, Carly Rosen, Gabrielle Siegel, Marcia Driscoll, Yvonne M Rasko

**Affiliations:** 1 Plastic Surgery, University of Maryland School of Medicine, Baltimore, USA; 2 Plastic and Reconstructive Surgery, University of Maryland School of Medicine, Baltimore, USA; 3 Surgery, University of Maryland School of Medicine, Baltimore, USA; 4 Medical Education, Lister Hospital, Stevenage, GBR; 5 Dermatology, University of Maryland School of Medicine, Baltimore, USA

**Keywords:** authorship, dermatology, residency and internship, bibliometrics, publications, h-index

## Abstract

Introduction

Research can be used to enhance the competitiveness of an application and is associated with a successful match. However, current reports regarding the publication record among prospective dermatology residents may be inaccurate. We sought to accurately assess the research credentials of matched dermatology residency candidates at the time of application.

Methods

We performed a bibliometric analysis to identify published articles of 1152 matched dermatology candidates and calculated the h-index of each applicant at the time of application. Details on article type, first authorship, and dermatology-relatedness of articles were collected.

Results

The median number of publications was two and the median h-index was 0. At the time of residency application, one-quarter of matched dermatology candidates (24%, n=278) possessed no publications. Over time, the median number of publications (R 0.10, p<0.001) and h-index (R 0.07, p=0.014) of matched applicants increased. The proportion of first-authored articles, dermatology-related papers, and each article type remained constant across application cycles (p>0.0500). An additional graduate degree, completion of a research fellowship, and graduation from a non-US medical school were independently associated with greater research credentials (p<0.0500).

Conclusions

Each year, applicants are publishing more articles and have a greater scholarly impact than in previous application cycles. However, the verified publication volume of matched dermatology applicants is strikingly lower than the values reported in national statistics.

## Introduction

Despite the expansion in the number of dermatology residency programs, the proportion of candidates failing to match in dermatology remains substantial [1-2], and it is becoming increasingly difficult to match into a dermatology residency position [3]. Multiple quantifiable factors play a role in candidate success, such as the United States Medical Licensing Examination (USMLE) scores, research productivity, and Alpha Omega Alpha (AOA) membership [4-6]. Indeed, 82% of dermatology programs require a minimum USMLE Step 1 score [7]. However, with the transition to a pass/fail score for Step 1 [8], program selection may rely more heavily on other factors such as publication count. Research can significantly impact the chance of a successful match [4] and remains one of the few quantifiable metrics that can be modified by applicants [9-10]. There is intense pressure on applicants to publish for the sake of improving their application and there have been reports of academic misrepresentation among dermatology applicants [11-12].

Previous studies and national data have reported on the research achievements of successful dermatology residency applicants [2-3,13-14]. However, they utilize self-reports, include abstracts and presentations within their publication count, and do not make a distinction between article subtypes, e.g. basic science versus clinical research content or systematic review versus case report. In addition, applicants may have different levels of contribution for each project as reflected by authorship status. However, to our knowledge, there is no literature on the peer-reviewed publication portfolio of successful dermatology applicants. Consequently, candidates may have a poor understanding of the strength of their research qualifications in comparison to other applicants. Reliable information on the research achievements of matched dermatology residency candidates is needed to properly inform prospective applicants and those who counsel them.

The purpose of this study is to use validated data sources to accurately assess the research credentials of successful dermatology residency candidates, as well as the variables independently associated with greater research credentials at the time of application. Additionally, we sought to clarify the extent of each candidate’s involvement in the published studies, the most prevalent article subtypes, and the relatedness of the publication to dermatology.

## Materials and methods

Setting and participants

This study was reviewed by the University of Maryland IRB and deemed to be exempt. We identified dermatology residency programs as listed by the Accreditation Council for Graduate Medical Education (ACGME) [15]. In September 2019, current residents for each program were identified by visiting the official websites of each residency program. We collected data for the dermatology residents in postgraduate years two to four, which corresponds to the 2015 to 2017 application cycles. We excluded all dermatology residents who applied for the residency match prior to 2015 or after 2017. Not all websites provided a list of their current residents. However, we collected details on 1152 dermatology residents, which is 87% (*n *= 1317) of the total matched population during the 2015 to 2017 application cycles [13-14]. We then collected details on gender, postgraduate year, additional degrees, prior research fellowship, and medical school through resident profiles available on individual program websites, LinkedIn (www.linkedin.com), and Doximity (www.doximity.com). Due to the use of multiple sources, we estimate this information to be close to 100% complete.

Outcomes

In 2019, we utilized three databases: Scopus (www.scopus.com), PubMed (www.ncbi.nlm.nih.gov/pubmed), and Google Scholar (https://scholar.google.com) to identify the peer-reviewed publications of each applicant. To account for publication lag, we included journal articles that were published on or before September of the first postgraduate year; i.e. for an individual who was successful in the 2017-2018 application cycle and then started residency training in July 2018, we included articles published in print on or before September 2018. This was done to be inclusive of articles listed as “accepted” as part of the application and reflect the information that would have been available in the match applications.

We collected the following research details for applicants at the time of application: 1) the total number of research publications; 2) h-index; 3) the number of first-authored papers; 4) the number of dermatology-related articles; and 5) the number of each article subtype (basic science studies, clinical research articles, literature review, systematic reviews and meta-analysis, case reports, book chapters, and editorial-type papers). Errata were excluded from publication counts. The dermatology-relatedness of the study was determined based on the specialty of the publishing journal and our review of the abstract. Editorial publications included letters to the editors, questions, commentaries, and editorials.

The primary outcome measures were the number of publications and h-index at the time of application. The h-index considers the number of publications and citations to calculate a score that measures an individual’s scholarly impact and does not merely reflect the academic output [16]. The h value is equal to the number of articles, “h”, that have been cited at least “h” times each. For example, author A has three publications that have been cited once, three, and eight times, respectively. Therefore, author A has an h-index of 2 because only two articles have at least two citations each. We manually calculated the h-index of each applicant at the time of application by reviewing the publication date of citing articles and only including those published before the candidate’s application.

Statistical analysis

Composite data were stored and analyzed in Microsoft Excel (2016, Microsoft Corporation, Redmond, Washington). The Kolmogorov-Smirnov test demonstrated that the number of publications and h-index did not follow a normal distribution. Therefore, these variables are summarized and analyzed using median values and interquartile ranges (IQR). We also reported mean values for the purpose of comparison to the National Resident Matching Program (NRMP) data. Linear regression was utilized to evaluate temporal trends in bibliometric data. To identify factors associated with an increase in the number of publications and h-index at application, variables were adjusted for collinearity and multivariate analysis was performed. Statistical significance was defined as a two-tailed value of p≤0.05.

## Results

We identified 1152 matched dermatology applicants for inclusion in this study. The characteristics of the cohort are described in Table 1. There was a total of 4804 publications, giving a mean of 4.2 articles per candidate.

**Table 1:** Characteristics of successful dermatology residency applicants Q1, lower quartile; Q3, upper quartile. a defined as the possession of a graduate degree (e.g. MA, MPH, MBA, Ph.D.) in addition to MD or DO degree

Characteristics of successful dermatology residency applicants				
	Applicants, No. (%)			
	Total	2015	2016	2017
Total	1152	372	390	390
Gender				
Male	468 (41)	137 (37)	170 (44)	161 (41)
Female	684 (59)	235 (63)	220 (56)	229 (59)
International medical graduate	28 (2)	5 (1)	12 (3)	11 (3)
Additional degree ^a^	142 (12)	47 (12)	47 (12)	48 (13)
Research fellowship	50 (4)	19 (5)	12 (3)	19 (5)

Publication details

The median number of publications was two (IQR: 1 - 6) and the median h-index was 0 (IQR: 0 - 1). At the time of residency application, one-quarter of the matched dermatology candidates (24%, n=278) possessed no publications. Moreover, over one third (36%, n=415) of successful dermatology applicants had not first-authored a paper. Clinical research articles were the most common publication subtype (26%, n=1250) and almost three-quarters of all publications held by applicants (72%, n=3435) were related to dermatology (Table 2). 

**Table 2:** Publication details of successful dermatology residency applicants Q1, lower quartile; Q3, upper quartile

Publication details of successful dermatology residency applicants				
	Publications			
Variable	Total No. (%)	2015 No. (%)	2016 No. (%)	2017 No. (%)
Total number of publications	4804	1276	1646	1882
Median number of publications (Q1, Q3)	2 (1, 6)	2 (0, 5)	3 (1, 5)	3 (1, 6)
Median h-index (Q1, Q3)	0 (0, 1)	0 (0, 1)	0 (0, 1)	1 (0, 2)
Number of publications first-authored by applicants	2372 (49)	651 (51)	792 (48)	929 (49)
Number of publications related to dermatology	3435 (72)	887 (70)	1199 (73)	1349 (72)
Article subtype				
Basic science	831 (17)	256 (20)	285 (17)	290 (15)
Clinical research	1250 (26)	327 (26)	384 (23)	539 (29)
Literature review	803 (17)	192 (15)	291 (18)	320 (17)
Systematic review and meta-analyses	169 (4)	32 (3)	55 (3)	82 (4)
Case reports	842 (18)	230 (18)	290 (18)	322 (17)
Book chapters	123 (3)	37 (3)	50 (3)	36 (2)
Editorial-type articles	785 (16)	201 (16)	292 (18)	292 (16)

Temporal analysis

**Figure 1:**
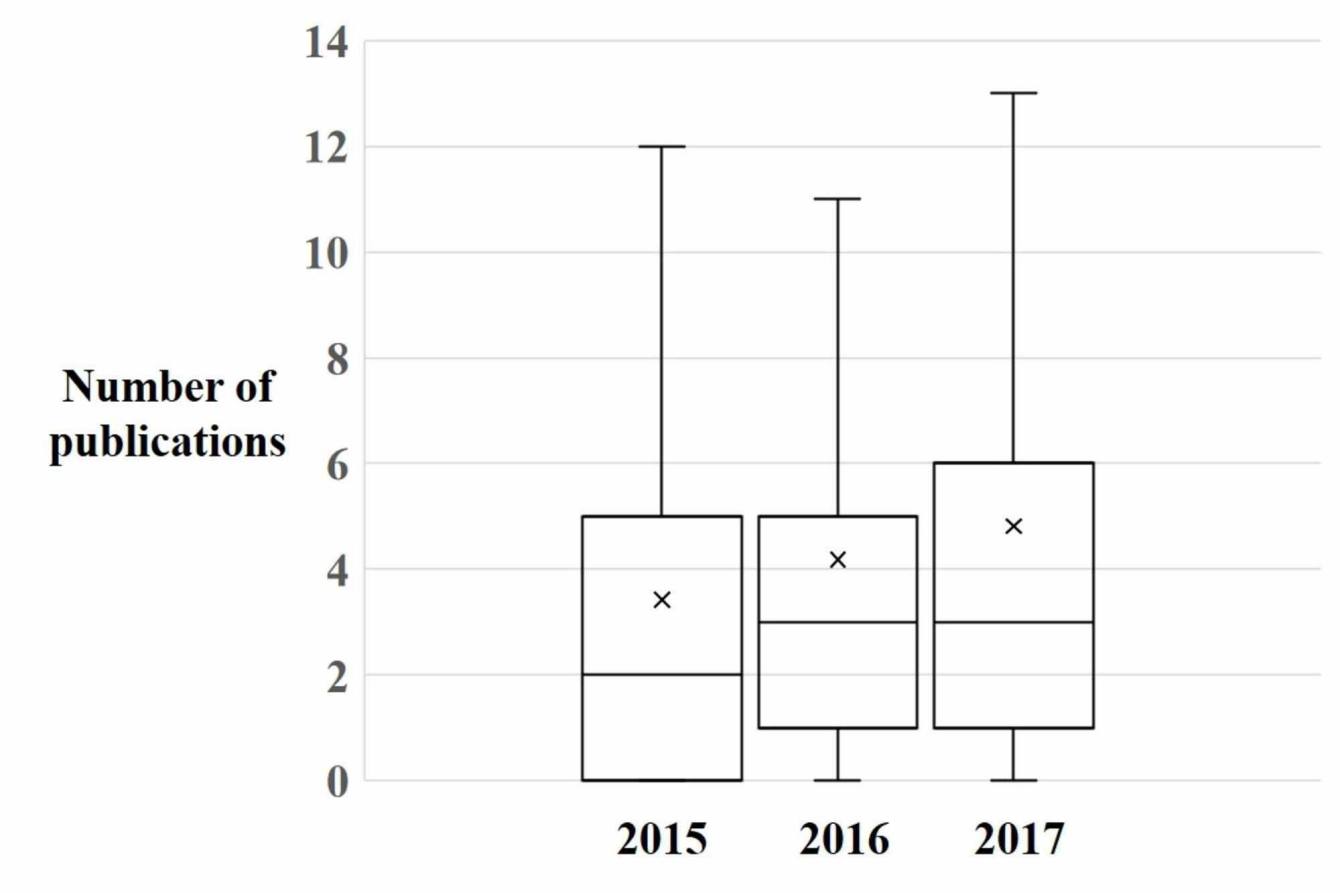
Publication volume of matched dermatology residents A boxplot demonstrating the number of publications held by matched dermatology candidates for each application year. X represents the mean value.

**Figure 2:**
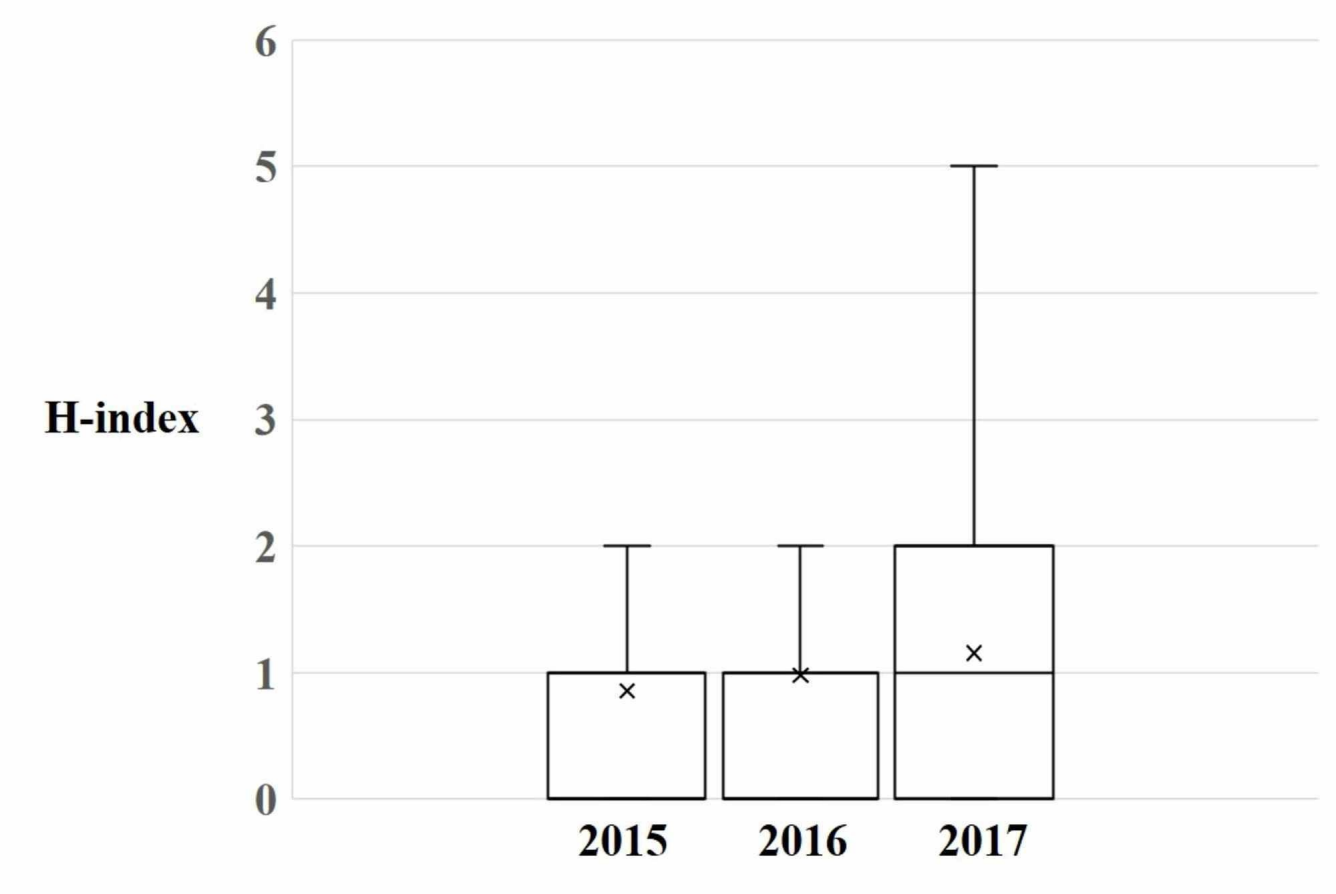
Scholarly impact of matched dermatology residents A boxplot demonstrating the h-index held by matched dermatology candidates for each application year. X represents the mean value.

Across application cycles, the median number of publications (R 0.10, p<0.001) (Figure 1) and h-index (R 0.07, p=0.014) (Figure 2) of matched applicants increased. The number of applicants without any publications declined but this was not significant (R -1.00, p=0.061). The number of applicants who had not first-authored any articles was unchanged (R 0.72, p=0.488). Similarly, the proportion of publications that were related to dermatology topics (R=0.01, p=0.707) remained constant. Additionally, the proportion of articles first-authored by applicants trended downwards over application cycles (R -0.06, p=0.075).

Of the articles subtypes, the proportion of basic science studies (R 0.03, p=0.422), literature reviews (R 0.05, p=0.182), systematic reviews and meta-analyses (R 0.03, p=0.446), case reports (R 0.03, p=0.367), and book chapters (R 0.02, p=0.620) remained consistent over time. The proportion of clinical research papers increased (R 0.06, p=0.066), and editorial-type articles deceased (R -0.06, p=0.088). However, this did not reach significance.

Multivariate analysis

Multivariate analysis demonstrated that completion of an additional graduate degree (β 0.13, p<0.001) and graduation from a medical school outside of the United States (β 0.05, p=0.040) were independently associated with an increase in applicant publication count. The proportion of dermatology-related articles held by a candidate was positively associated with more publications (β 0.12, *p*=0.004). Additionally, all articles subtypes were positively associated with an increase in the number of publications; clinical research articles (β 0.24, p<0.001), literature reviews (β 0.23, p<0.001), and editorial-type papers (β 0.23, p<0.001) were the most strongly associated article subtypes (Table 3). 

**Table 3:** Multivariate analysis of factors associated with an increase in the number of publications at the time of dermatology residency application * denotes statistical significance. a defined as the possession of a graduate degree (e.g. MA, MPH, MBA, Ph.D.) in addition to MD or DO degree.

Variable	Effect Coefficient (β)	95% Confidence Interval	p-value
Applicant characteristic			
Application year	0.05	-0.10 - 0.00	0.057
Additional degree ^a^	0.13	0.08 - 0.19	*<0.001
Research fellowship	0.03	-0.02 - 0.08	0.231
International medical graduate	0.05	0.00 - 0.10	*0.040
Publication details			
Proportion of publications first-authored by applicants	-0.07	-0.15 - 0.00	0.055
Proportion of publications related to dermatology	0.12	0.04 - 0.20	*0.004
Article subtype			
Proportion of basic science studies	0.21	0.15 - 0.27	*<0.001
Proportion of clinical research articles	0.24	0.18 - 0.31	*<0.001
Proportion of literature reviews	0.23	0.16 - 0.29	*<0.001
Proportion of systematic reviews	0.14	0.08 - 0.19	*<0.001
Proportion of case reports	0.11	0.03 - 0.19	*0.009
Proportion of book chapters	0.10	0.04 - 0.15	*<0.001
Proportion of editorial-type articles	0.23	0.16 - 0.30	*<0.001

An additional graduate degree (β 0.21, p<0.001), research fellowship (β 0.06, p=0.019), and graduation from a non-US medical school (β 0.23, p=0.001) were independently associated with an increasing h-index. Publication of any article subtypes, with the exception of book chapters, was associated with an increase in h-index. Notably, basic science studies had the strongest association with h-index (β 0.47, p<0.001) (Table 4). 

**Table 4:** Multivariate analysis of factors associated with an increasing h-index at the time of dermatology residency application * denotes statistical significance. a defined as the possession of a graduate degree (e.g. MA, MPH, MBA, Ph.D.) in addition to MD or DO degree.

Variable	Effect Coefficient (β)	95% Confidence Interval	p-value
Applicant characteristic			
Application year	0.04	-0.09 - 0.24	0.147
Additional degree^ a^	0.21	0.16 - 0.26	*<0.001
Research fellowship	0.06	0.01 - 0.10	*0.019
International medical graduate	0.08	0.04 - 0.13	*0.001
Publication details			
Proportion of publications first-authored by applicants	-0.01	-0.08 - 0.06	0.740
Proportion of publications related to dermatology	-0.07	-0.14 - 0.00	0.067
Article subtype			
Proportion of basic science studies	0.47	0.41 - 0.52	*<0.001
Proportion of clinical research articles	0.28	0.22 - 0.34	*<0.001
Proportion of literature reviews	0.17	0.10 - 0.23	*<0.001
Proportion of systematic reviews	0.09	0.03 - 0.14	*0.001
Proportion of case reports	0.08	0.00 - 0.16	*0.038
Proportion of book chapters	0.03	-0.02 - 0.08	0.180
Proportion of editorial-type articles	0.11	0.05 - 0.17	*<0.001

## Discussion

As matching into dermatology residency programs becomes more difficult each year [3], it becomes important to accurately characterize the scholarly profile of successful dermatology residency applicants. We hope this detailed report of the scholarly profiles of matched dermatology residency applicants will better inform future applicants and those who advise them. Our results demonstrate that 1) the average number of publications held by successful candidates is lower than values reported in national statistics; 2) each year, applicants are publishing more articles than in previous application cycles; 3) the scholarly impact (h-index) of matched dermatology candidates is growing; and 4) an additional graduate degree, research fellowship completion, and graduation from a non-US medical school were predictors of greater research credentials.

It is important to note that the mean number of research publications found in this study (4.2) differed by a factor of three from values reported by the NRMP (14.7) [13]. This discrepancy is consistent with the observations of other competitive medical specialties such as plastic surgery [17], neurosurgery [18], and otolaryngology [19]. This inconsistency may be explained by the flaws within NRMP data collection methods: (i) it is self-reported; (ii) inclusion of abstracts, oral presentations, and posters within the publication count; (iii) failure to include data from all successful candidates; and (iv) inclusion of submitted papers. Furthermore, the skewness of the data necessitates the use of median values instead of the mean value employed by NRMP. Consequently, the national data may be misleading when it comes to the number of peer-reviewed articles held by successful dermatology residency applicants. Medical students wishing to apply to dermatology should be advised that while the average matched applicants hold two published or accepted articles at the time of application, this number is trending upwards over time.

To our knowledge, we are the first paper to report on the h-index of matched dermatology residency applicants. The relatively low median h-index may reflect an applicant's limited research experience, as medical students may be more likely to publish in lower impact journals and possess short research careers. Our study found that matched dermatology candidates hold increasing research accomplishments each year, which is consistent with trends seen in the literature [2-3,13-14]. The rising h-index of successful candidates may indicate that not only quantity but the quality of publications is increasing. Given the growing competitiveness of a dermatology residency position [1,3] and the association between the number of publications and successful matching into dermatology [9-10], it is unsurprising that applicants are increasing their scholarly credentials in the hopes of improving their chances of success. Moreover, the proportion of first-authored articles declined as the total number of publications per each applicant increased. This may indicate that as students strive to enhance their academic productivity by taking on multiple projects, they sacrifice the responsibility and ownership of one project. Alternatively, there may be an overall increased interest in research in recent years. It is important to remember that the escalating pressure to publish may increase the incidence of academic misrepresentation [11-12]. Peer-reviewed publications are significantly preferred by dermatology program directors over oral presentations, poster presentations, and abstracts [6]. Moreover, applicants who list several unpublished manuscripts have greater odds of matching, even if the manuscripts remain unpublished [9]. Therefore, publication inflation through the listing of multiple “submitted” manuscripts may be an alternative cause of the discrepancy between NRMP-reported data and the results of this study.

Additional graduate degrees, research fellowship, and graduation from a medical school overseas were independently associated with increased research qualifications. It is well-established that international medical graduates can improve their chances of a successful match with a higher number of publications [20] and consistently demonstrate greater academic credentials than their US peers [17]. Research fellowship and advanced degrees, which are factors within the control of the applicant, have been associated with increased scholarly productivity [17-18,21]. Mentors and applicants can consider the potential benefits of a research fellowship or combined degree programs, such as an MD/MPH, MD/Ph.D., or MD/MBA program. Although these factors may improve the chances of a favorable dermatology residency match, they cannot guarantee success, as research productivity is only one component of the selection criteria and other facets of an application also influence decision-making such as clerkships scores, USMLE scores, and interviews. To strengthen their candidate profile, medical students interested in dermatology should consider all aspects of their application.

Our multivariate analysis found that certain publication subtypes had greater associations with scholarly productivity and impact. Clinical research studies, literature reviews, and editorial-type articles are often viewed as less time-consuming to set up and faster to publish, which may explain their ability to enhance an applicant’s publication volume. Whereas, original research articles (basic science studies and clinical research papers) are cited more frequently in comparison to other publication subtypes, such as case reports [22], which results in an increase in h-index. Given our findings, future applicants who wish to enhance their scholarly portfolio and research proficiency can consider engaging in clinical research and basic science studies, which may enhance both publication count and scholarly impact.

There are limitations to this study. First, data on unsuccessful dermatology candidates are not available, which prevents the comparison of these two cohorts. Future investigations comparing the research productivity and profiles of matched and unmatched candidates are needed to evaluate the true impact of research on match success. Second, we utilized online resources to collect data. Websites may have been outdated, have incomplete resident profiles, or included an incomplete list of residents. However, we collected data on 87% of the total dermatology residency applicants [13-14], making our results a close representation of the current cohort. Furthermore, we utilized multiple online resources to confirm details on the candidates so we believe the rate of errors to be low. Third, we were not able to collect details on abstracts and presentations, both of which form part of the research profile of a dermatology applicant. Lastly, a flaw of the h-index scoring, in that it can be artificially elevated through self-citation. Given the short academic career of medical students, the number of self-citations is likely limited and unlikely to account for the observed rise in h-index.

## Conclusions

The verified publication volume of matched dermatology applicants is notably lower than values reported in national statistics. Each year, applicants are publishing more articles and have a greater scholarly impact than in previous application cycles. The completion of a research fellowship or advanced degree is associated with higher research productivity.
